# Molecular profile and copy number analysis of sporadic colorectal cancer in Taiwan

**DOI:** 10.1186/1423-0127-18-36

**Published:** 2011-06-07

**Authors:** Chien-Hsing Lin, Jen-Kou Lin, Shih-Ching Chang, Ya-Hui Chang, Hwey-May Chang, Jin-Hwang Liu, Ling-Hui Li, Yuan-Tsong Chen, Shih-Feng Tsai, Wei-Shone Chen

**Affiliations:** 1 Division of Molecular and Genomic Medicine, National Health Research Institutes, Zhunan, Taiwan; 2 Division of Colon and Rectal Surgery, Department of Surgery, Taipei Veterans General Hospital, Taipei, Taiwan; 3 Division of Hematology and Oncology, Taipei Veterans General Hospital, Taipei, Taiwan; 4 Institute of Biomedical Sciences, Academia Sinica, Taipei, Taiwan; 5 Genome Research Center and Department of Life Sciences and Institute of Genome Sciences, National Yang-Ming University, Taipei, Taiwan

## Abstract

**Background:**

Colorectal cancer (CRC) is a major health concern worldwide, and recently becomes the most common cancer in Asia. The case collection of this study is one of the largest sets of CRC in Asia, and serves as representative data for investigating genomic differences between ethnic populations. We took comprehensive and high-resolution approaches to compare the clinicopathologic and genomic profiles of microsatellite instability (MSI) *vs*. microsatellite stability (MSS) in Taiwanese sporadic CRCs.

**Methods:**

1,173 CRC tumors were collected from the Taiwan population, and sequencing-based microsatellite typing assay was used to determine MSI and MSS. Genome-wide SNP array was used to detect CN alterations in 16 MSI-H and 13 MSS CRCs and CN variations in 424 general controls. Gene expression array was used to evaluate the effects of CN alterations, and quantitative PCR methods were used to replicate the findings in independent clinical samples.

**Results:**

These 1,173 CRC tumors can be classified into 75 high-frequency MSI (MSI-H) (6.4%), 96 low-frequency MSI (8.2%) and 1,002 MSS (85.4%). Of the 75 MSI-H tumors, 22 had a *BRAF *mutation and 51 showed *MLH1 *promoter hypermethylation. There were distinctive differences in the extent of CN alterations between CRC MSS and MSI-H subtypes (300 Mb *vs*. 42 Mb per genome, *p*-value < 0.001). Also, chr7, 8q, 13 and 20 gains, and 8p and 18 losses were frequently found in MSS but not in MSI-H. Nearly a quarter of CN alterations were smaller than 100 kb, which might have been missed in previous studies due to low-resolution technology. 514 expressed genes showed CN differences between subtypes, and 271 of them (52%) were differentially expressed.

**Conclusions:**

Sporadic CRCs with MSI-H displayed distinguishable clinicopathologic features, which differ from those of MSS. Genomic profiling of the two types of sporadic CRCs revealed significant differences in the extent and distribution of CN alterations in the cancer genome. More than half of expressed genes showing CN differences can directly contribute to their expressional diversities, and the biological functions of the genes associated with CN changes in sporadic CRCs warrant further investigation to establish their possible clinical implications.

## Background

Colorectal cancer (CRC) is one of the major leading causes of cancer deaths around the world, and is the most common cancer in Taiwan [1]. Two different genetic pathways have been described for tumorigenesis of CRC. The most frequent pathway is the chromosomal instability pathway characterized by alterations in tumor suppressor genes and oncogenes, including *APC*, *TP53 *and *K-ras *[2,3]. On the other hand, 10-15% of all cases of CRC show microsatellite instability (MSI), which are resulted from a germline mutation in the mismatch repair (MMR) system or somatic hypermethylation of the promoter region of the *MLH1 *gene [4]. Tumors with MMR deficiency exhibited frequent errors in microsatellite DNA, short segments of DNA containing tandem repeats of mono-, di- or trinucleotides [5]. The high-frequency MSI (MSI-H) CRCs have unique clinicopathologic features, such as right-sided, mucinous or poorly differentiated, and stable chromosomal status in the tumors [6].

About 80% of MSI tumors have a near-diploid karyotype and a distinct genetic alteration distinguishable from those of microsatellite stable (MSS) cancers [7-10]. Despite the advancement of our understanding of cancer genetics of CRC, genomic alterations of various subtypes of CRC have not been fully characterized. The copy number variations (CNVs) can contribute to variable levels of gene expressions [11], and thus fine-scale copy number (CN) profiling of cancer can enhance our knowledge about tumorigenesis. Among all somatic mutations, non-germline CNVs found in the cancer genomes, also known as copy number alterations/aberrations (CNAs), are frequently observed, e.g., gains of oncogenes and losses of tumor suppresser genes [12]. Furthermore, the DNA CN states of CRC cases are related to the response of drug treatments, e.g., the CNA degree of CRC is associated with response to systemic combination chemotherapy with capecitabine and irinotecan [13].

Previous cytogenetic studies have shown MSS tumors are characterized with more chromosomal and copy number aberrations than MSI tumors [14,15], and most of MSI tumors have a near-diploid karyotype and appear to follow a genetic pathway distinct from MSS tumors [9]. These studies showed that gain of chromosome 7, 8q, 13 and 20q and loss of chromosome 4q, 8p, 17p and 18q were frequent in CRC MSS tumors [16]. Both profiles of genome-wide CNA and gene expression have been used to classify MSS and MSI subtypes of CRC samples [17]. However, previous genome-wide CNA studies of CRC were limited by the resolution of comparative genomic hybridization (CGH) array technology (probe distance > 30 kb), thereby subtle CN changes harboring cancer-causing variants might be missed [13,17,18]. As genomic technology advances, high-density single-nucleotide polymorphism (SNP) array can be used to genotype a huge number of SNPs and detect CN changes on the genomic scale. In the current study, we have applied Affymetrix SNP 6.0 array (Affymetrix, CA, USA), with its median inter-probe distance of less than 700 bp, to detect CNAs in CRC cancer genome of clinical samples. As compared to other reports on the CRC cancer genome using the CGH arrays, we have achieved a much improved resolution. Molecular karyotype profiling of the two subtypes of sporadic CRCs revealed significant differences in the extent and distribution of CN alterations in the cancer genome. Combining the data of genome-wide CNAs and Illumina Human Ref-8 gene expression array (Illumina, CA, USA), CNAs might significantly contribute to the expressional levels of genes, more than half of which were differently expressed between CRC MSI-H and MSS.

## Materials and methods

### Clinical patients and tumor tissues

A total of 1,543 colorectal cancer patients who underwent surgeries in Taipei Veterans General Hospital from January 2000 to December 2007 were included. The study was approved by the Institutional Review Board of the Taipei Veterans General Hospital, and written informed consent for tissue collection was obtained from all patients. Patient with preoperative chemoradiotherapy, or emergent operative procedure, or death within 30 postoperative days, or evidence of familial adenomatous polyposis were excluded from this study. Clinical information was recorded prospectively and stored in a database. This included: (i) age, sex, personal and family history, and (ii) tumor size, location, gross appearance, TNM stage, differentiation and pathological prognostic features. Tumors were meticulously dissected, with samples collected from the 4 tumor quadrants to explore intratumoral heterogeneity. The corresponding normal mucosa, at least 10 cm away from the primary tumor edge, was collected. Tissue fragments were immediately frozen in liquid nitrogen and stored at -70°C. Sections of cancerous and collateral tissues were reviewed and analyzed by a senior gastrointestinal pathologist blinded to patient outcomes. Disease stage was determined with the TNM classification of the International Union Against Cancer [19]. The pathological factors analyzed included lymphovascular invasion, invasive tumor pattern, grade of differentiation, mucin production and intratumoral lymphocyte infiltration. These pathological features were defined by the College of American Pathologists consensus statement [20].

### Microsatellite Instability Analysis

High-molecular-weight genomic DNA from each tumor and from corresponding normal tissue was purified using the QIAamp Tissue kit (QIAGEN GmbH, Germany). Yield and purity were determined by electrophoresis on 0.8% agarose gel and spectrophotometric absorbance at 260 nm. According to international criteria for determination of MSI,^5 ^five reference microsatellite markers were used: *D5S345*, *D2S123*, *BAT25*, *BAT26*, and *D17S250*. Primer sequences were obtained from GenBank (http://www.gdb.org). Detection of MSI was performed as previously described [20,21]. Briefly, DNA was amplified using fluorescent polymerase chain reaction (PCR). PCR products were denatured and analyzed by electrophoresis on 5% denaturing polyacrylamide gels, and results were analyzed using GeneScan Analysis software (Applied Biosystems, CA, USA). Tumor samples that exhibited allele peaks different from the corresponding normal sample(s) were classified as MSI for that particular marker. Samples with ≥ 2 MSI of 5 markers were defined as MSI-H, those with only one MSI of 5 markers were defined as low-frequency MSI (MSI-L) and others without evidence of MSI were classified as MSS. Analyses were performed twice if results were ambiguous.

### Immunohistochemistry

Immunohistochemistry (IHC) staining for MLH1, MSH2, MSH6 and PMS2 were done for cases with MSI-H. Paraffin-embedded tissue sections (4 μm thickness) were stained with antibodies for MLH1 (1:10 dilution, Pharmingen), MSH2 (1:200, Oncogene Research Products), MSH6 (1:300, Transduction Laboratories) and PMS2 (C20) (1:400, Santa Cruz Biotechnology). Negative control slides were made without the primary antibody.

### *BRAF *mutation and *MLH1 *methylation analysis

To detect *BRAF *mutation, DNA from tumor tissue was amplified and sequenced with primers described in previous studies [22]. Briefly, the extracted DNA was selectively amplified by PCR in a DNA thermocycler. A negative control containing no DNA template was included for each PCR amplification round. The PCR products were analyzed by an automated sequencer (ABI Prism 3100 Genetic Analyzer; Applied Biosystems). Each sample was sequenced on both sense and antisense strands. Each mutation was confirmed by a second sequencing procedure on new PCR products. Methylation of the *MLH1 *promoter was determined using a methylation-specific PCR method. DNA was treated with sodium bisulfite, which converts unmethylated cytosine to uracil, yet leaves methylated cytosine unchanged, and subjected to amplification with methylated- and unmethylated-specific primers, respectively [23].

### Flow Cytometry for DNA Ploidy

703 of 1,173 tumors were available to examine the status of DNA ploidy using flow cytometry by following the method of Dressler *et al*. [24]. The DNA index (DI), representing the ratio of the mean fluorescence intensity of the G_0_G_1 _peak of the tumor cell population to that of the normal diploid population, was used to quantitate DNA ploidy. Specimens were considered diploid (DI = 1) if they had a single G_0_G_1 _peak and aneuploid (DI ≠ 1) if they exhibited two or more discrete peaks, including abnormal G_0_G_1 _peaks (each peak equivalent to the fluorescence of at least 20% of the total sample nuclei) and a corresponding G_2_M peak. Samples with coefficients of variation > 8% were excluded from further analysis [21]. Tumors with both diploid and aneuploid subpopulations were classified as having DNA aneuploidy. The mean coefficients of variation were 6.4% and 2.4% in tumor tissues and normal colon mucosa, respectively.

### High-density SNP array and data analysis

A total of 500 ng of genomic DNA of 16 MSI-H and 13 MSS CRC samples was subjected to SNP genotyping using genome-wide Affymetrix Human SNP 6.0 array according to the manufacturer's instructions. Genotyping was performed by the National Genotyping Center at Academia Sinica, Taipei, Taiwan (http://ngc.sinica.edu.tw). This array contains 1.8 millions markers widely distributing in human genome. After standard Affymetrix quantile normalization, the intensity data was analyzed using Genotyping Console (GTC) software v.3.0.1 (Affymetrix) with default parameters of hidden-Markov model (HMM) to identify CN-changed regions [25]. PennCNV [26] and Partek Genome Suite (Partek Inc., MO, USA) software were additionally used to reconfirm CN alterations identified by GTC software. CNA predicted by PennCNV and Partek software with default HMM parameters are 91.6% and 89.8% concordant with those of GTC software. In consideration of CN-changed regions with at least 20 consecutive probes, we found that all these CNA identified are 100% overlapped with those defined by either PennCNV or Partek software, implying these CNAs were highly reliable for the following analysis.

### Quantitative genomic PCR

CN changes of selected genes, including epidermal growth factor receptor (*EGFR*), deleted colon cancer (*DCC*) and calcium-dependent membrane-binding protein 1 (*CPNE1*), were verified by using quantitative genomic PCR experiments. Primer Express Software version 3.0 (Applied Biosystems) was applied to design PCR primers for the selected target genes. Quantitative genomic PCR were performed using the ABI StepOne Plus system (Applied Biosystems). PCR reactions were prepared using the Power SYBR-Green PCR reagent kit (Applied Biosystems), and 2.5 ng genomic DNA was used in each reaction. qPCR conditions were as follows: initial denaturation at 94°C for 3 minutes, followed by 40 cycles of denaturation at 94°C for 15 seconds, and combined annealing and extension at 60°C for 60 seconds. The fluorescence signal was detected in real time during the qPCR procedure. The primer pair for the long interspersed nuclear elements 1 sequence was used for normalization. The mean estimated CN was calculated from triplicate PCR reactions for each individual.

### Whole-genome gene expression analysis

RNA samples of 16 MSI-H and 13 MSS tumors (identical cases as used in SNP array analysis) were prepared using Qiagen's RNAeasy kit (Qiagen), and then were assayed using the Agilent Systems Bioanalyzer (Agilent Technologies, CA, USA) to ensure that high-quality RNA was used for the gene expression array experiments. The Illumina TotalPrep RNA amplification kit (Ambion, TX, USA) was used to amplify and generate biotinylated RNA. Illumina Human Ref-8 V3 arrays were processed and scanned at medium PMT settings as recommended by the manufacturer, and were analyzed using GenomeStudio software (Illumina). After subtracting background, array data was normalized using the quantile method, and detection p-value < 0.01 was used to ensure that only expressed genes were used in the following analyses.

### Statistical analysis

All results in the text and tables are given as means ± standard deviation. In clinical analyses, categorical variables were analyzed using a chi-square test with Yates' correction, and comparisons of quantitative variables between groups were performed based on Student's *t*-test. In genomic data analysis, CNA frequency comparisons between CRC MSS and MSI-H subtypes were carried out by using Fisher's exact test, and t-test was applied in comparing expressional levels of each transcript between CRC subtypes. SAS/STAT (SAS Institute, NC, USA) program was used to carry out all statistical analyses.

## Results

**Figure 1:**
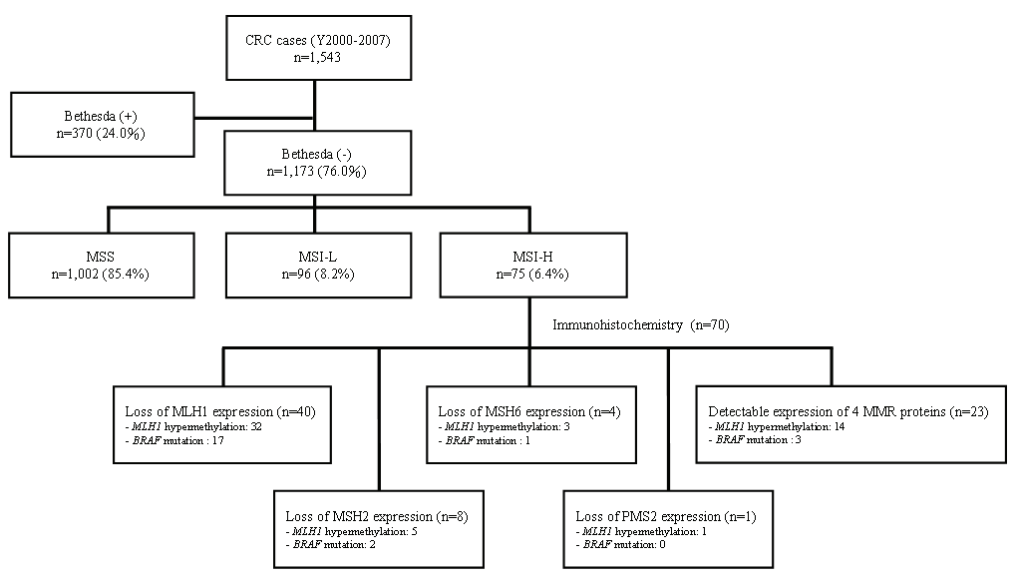
**Flowchart of genomic study on sporadic CRCs**. Five reference microsatellite markers are used to classify sporadic CRC cases into microsatellite stability (MSS), low-frequency microsatellite instability (MSI-L), and high-frequency MSI (MSI-H) (shown in *Materials and Methods*). Immunohistochemistry staining for MLH1, MSH2, MSH6 and PMS2 protein and mutation screening for *BRAF *gene were done for CRC cases with MSI-H.

A total of 1,543 CRCs were recruited in Taiwan population from 2000 to 2007 as shown in Figure 1. To focus on sporadic CRC cases for the clinicopathologic and genomic analyses, 370 (24.0%) meeting the Revised Bethesda criteria [27], defined patients having CRC familiar history, were excluded, and the remaining 1,173 patients were sporadic CRC cases. There were 785 (66.9%) males and 388 (33.1%) females in these sporadic CRC patients. Tumors were found in right-side colon in 294 patients (25.1%), left-side colon in 478 patients (40.8%), and in the rectum in 401 patients (34.2%). There were 159 patients (13.6%) with stage I cancers, 395 patients (33.7%) with stage II cancers, 407 patients (34.7%) with stage III cancers and 212 patients (18.1%) with stage IV cancers. Based on microsatellite instability analysis, among the 1,173 tumors analyzed, 75 (6.4%) were MSI-H, 96 (8.2%) were MSI-L, and 1,002 (85.4%) were MSS. Interestingly, 48 out of the 75 MSI-H tumors (64%) were located in the right colon; 67% had stage I or II disease; 60% were female and 24% were poorly or mucinous differentiated (Table 1). In contrast to the clinopathologic features of MSI-H tumors, MSS/MSI-L showed left sided predominant, less mucinous or poorly differentiation and more advanced disease.

**Table 1:** Clinico-pathological differences between MSI-H and MSI-L/MSS CRCs

Variables	MSI-H tumors (N = 75)	MSI-L/MSS tumors (N = 1,098)	p-value
Age	70.2 ± 9.6	70.8 ± 9.2	0.565
Female gender (%)	45(60)	343(31.2)	< 0.001
Right colon (%)	48(64.0)	246(22.4)	< 0.001
Stage 1,2 (%)	50(66.7)	504(45.9)	< 0.001
Mucinous or signet ring adenocarcinoma (%)	18(24.0)	112(11.0)	0.001
Poor differentiated (%)	18(24.0)	57(5.2)	< 0.001

Categorical variables were analyzed using a chi-square test with Yate's correction.

Comparisons of quantitative variables between groups used Student's *t*-test.

**Figure 2:**
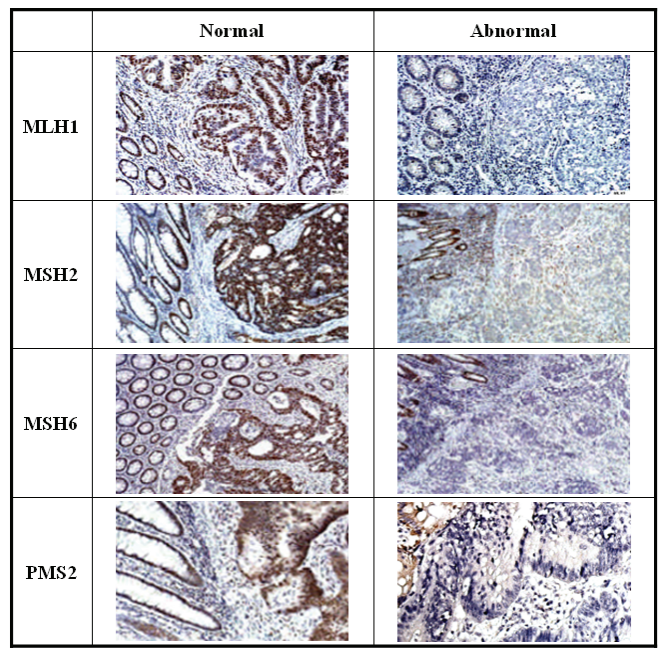
**Immunohistochemical (IHC) stains for MLH1, MSH2, MSH6 and PMS2 proteins**. Paraffin-embedded tissue sections (4 μm thickness) of CRC MSI-H and control samples were stained with antibodies for MLH1, MSH2, MSH6 and PMS2 proteins.

Methylation of the *MLH1 *gene promoter and *BRAF *gene mutations were analyzed for all MSI-H tumors. Of the 75 MSI-H tumors, 22 (29.3%) had a *BRAF *mutation and 51 (68%) showed hypermethylation of the *MLH1 *gene promoter. Immunohistochemical (IHC) stains for MLH1, MSH2, MSH6 and PMS2 proteins were carried out for 70 cases with MSI-H tumors whose samples were available (Figure 2). As shown in Figure 1, 47 of 70 (67.1%) MSI-H tumors showed abnormalities with IHC analysis for at least one MMR protein. The majority (n = 40, 57.1%) lost MLH1 protein expression, followed by MSH2 protein (n = 8, 11.4%). Among the 40 tumors with no detectable MLH1 protein expression, 32 had hypermethylation of the promoter (80%) and 17 had *BRAF *mutation (42.5%). Five MSI-H tumors had no expression of either MSH6 or PMS2 protein, and 23 cases (32.9%) had detectable expressions of all four MMR proteins (Figure 1).

**Figure 3:**
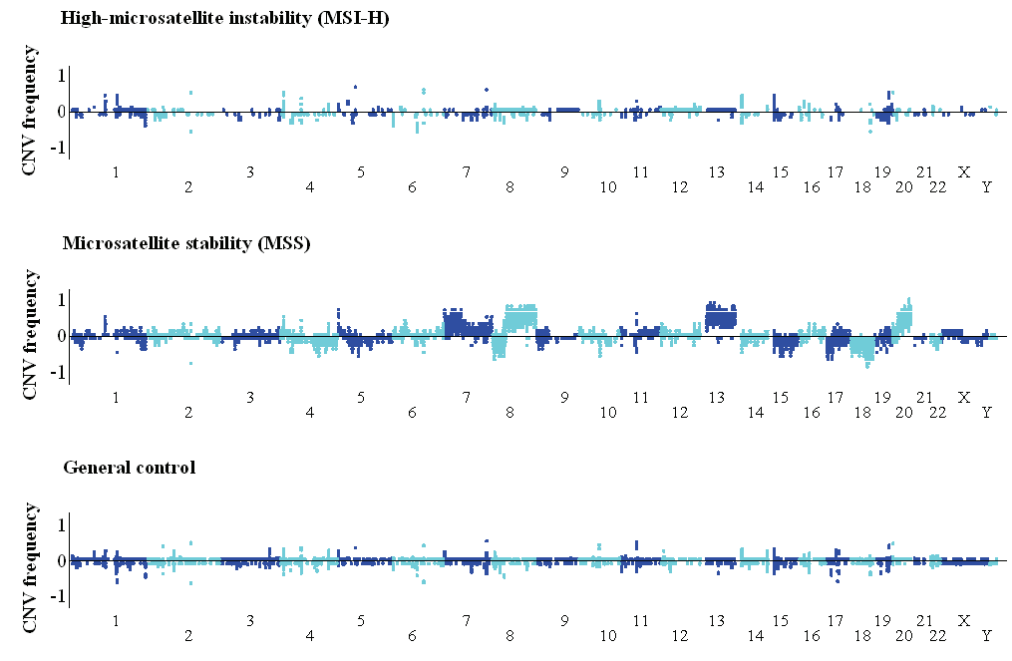
**Whole-genome copy number variation (CNV) pattern of colorectal cancer and general population**. The CNV frequencies are measured from 16 MSI-H CRCs, 13 MSS CRCs and 434 individuals from general population using Affymetrix SNP 6.0 array. Top dots represent the frequencies of CN gains, and bottom dots represent the frequencies of CN losses.

**Figure 4:**
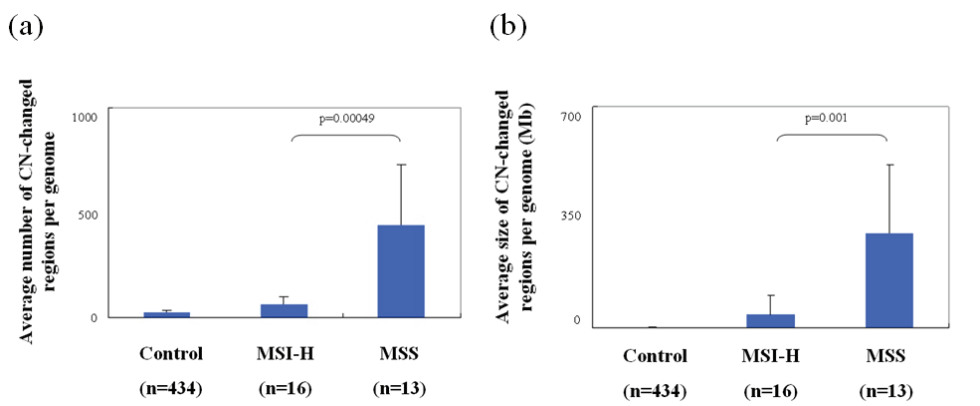
**Comparisons of copy number variation patterns between colorectal cancer subtypes**. (a). the average number of CN-changed regions per genome for MSS, MSI-H and general controls. (b). the average size of CN-changed regions per genome for MSS, MSI-H and general controls.

Of the 703 tumors, including 51 MSI-H and 652 MSI-L/MSS, available for the status of DNA ploidy, 231 showed DNA diploid (32.9%). We found that 70.2% of MSI-L/MSS tumors showed DNA aneuploidy, but only 27.5% of MSI-H tumors showed DNA aneuploidy. To molecularly characterize chromosomal aberrations at a high resolution (≤ 20 kb) and compare the genomic features between the MSI-H and MSS subtypes, Affymetrix SNP 6.0 array was applied to detect genome-wide CNAs in 16 MSI-H tumors with both *MLH1 *hypermethylation and *BRAF *mutation, and compared to the genomic profiles of 13 MSS CRC tumors. To identify reliable CN changes, we only included CN-changed regions covering more than 20 probes, and these CNAs were also called by PennCNV and Partek CNV calling software (algorithm-independent). As a control, the CNV profile of Taiwanese population was based on 434 general controls from Han Chinese Cell and Genome Bank that were genotyped using Affymetrix SNP 6 array [28]. This data provides useful information, at the population scale, the common variation of genomic structure in the Taiwanese study subjects. A total of 399 CNV regions were identified in this population (Dr. Y.-T. Chen, *unpublished data*), the average size of the CNV regions was 350 kb (covering a total of 4.66% of the human genome), and 372 (93.23%) were reported in the database of genomic variants (http://projects.tcag.ca/variation/). As shown in Figure 3, the whole-genome CNV patterns of the two CRC subtypes were grossly different. DNA CN gain in chr7, 8q, 13 and 20 and loss in chr4q, 8p and 18 were frequently found in MSS but not in MSI-H tumors. Consisting with previous studies, the chromosomal structures of CRCs with microsatellite instability were similar to those of normal controls [9] (Figure 3). There were distinctive differences in the number of CNAs between CRC MSS and MSI-H subtypes (Figure 4a, 439 *vs*. 63 per genome, p-value = 0.0005), and the average size of CNAs per genome of MSS tumor was larger than that of MSI-H tumor (Figure 4b, 300 Mb *vs*. 42 Mb, p-value = 0.001).

**Figure 5:**
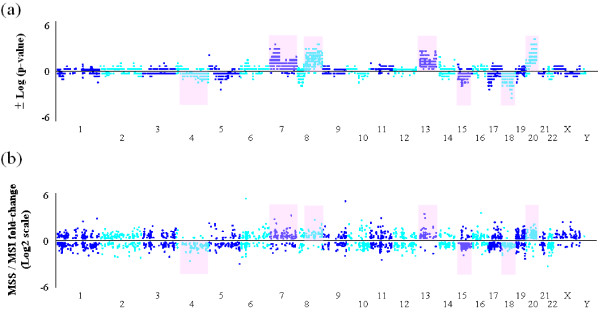
**Genomic profile comparisons between colorectal cancer (CRC) subtypes**. (a). Gene-based copy number alteration (CNA) frequency difference between CRC subtypes. Each dot represents the significance of CNA frequency difference between MSS and MSI-H subtypes of each gene (Fisher's exact test). Top dots indicate the -log_10 _(p-value) of genes with CN gains, and bottom dots indicate the log_10 _(p-value) of genes with CN losses. (b). Comparison of gene expression differences between CRC subtypes. Each dot represents the log2 scale of average expression fold-change (MSS/MSI-H) of each gene (two sample *t*-test, p-value < 0.05).

The majority of CNAs (> 80%) found in CRC cases was smaller than 500 kb, and nearly a quarter of CN alterations were smaller than 100 kb, which might have been missed in the previous studies due to low-resolution technologies (Additional File 1). Therefore, CNA frequencies of some DNA segments in this study were higher than those from previous studies (14). 13,279 protein-coding genes and 557 microRNA were affected by CN changes in these CRC samples, of which 1,434 genes (10.8%) and 35 microRNAs (6.3%) were related to CNVs observed in the general Taiwanese population. To identify genes harboring the CRC subtype-common and/or specific CN changes, the gene-based CNA frequency of MSS and MSI-H subtypes were compared as shown in Figure 5a. 1,515 of 13,279 genes (11.4%) were found to have CN frequency difference between MSS and MSI-H tumors using Fisher's exact tests (p-value < 0.05, Additional File 2), and CNA frequencies of these genes in MSS tumors were all higher than those in MSI-H tumors.

The CN gain of *EGFR *gene, a well-known cancer gene and drug target, was commonly found in CRC MSS tumors (8 out of 13 samples, 62%) according to genome-wide CNA analysis. To replicate the findings from the SNP array analysis, we applied qPCR approach to evaluate the *EGFR *CN states of independent 48 CRC MSS and 48 MSI-H samples (Additional File 3). The CN gain frequency of the independent CRC MSS group was 64.6% (31 of 48) and consisted to that (62%) of the array-based CN analysis, and was higher than overall 14% of CRC MSI-H subtype (n = 64). Furthermore, although CN losses of *DCC *gene were commonly found in CRCs in previous studies [29], we observed that this DCC deletions were frequently found in MSS CRCs (46%) but not in MSI-H (0%). Twelve cancer-associated genes were found to show different CN frequencies between CRC subtypes as shown in Table 2 (Fisher's exact test, p-value < 0.01), but the biological functions of many identified genes with high CNA frequencies were not fully characterized.

**Table 2:** Cancer genes showing differences in copy number aberration between CRC subtypes.

**Gene Symbol**^**1**^	Frequency of CN Gain			Frequency of CN Loss			Gene expression profile		
	
	**MSS**^**2**^	**MSI-H**^**2**^	P-value	**MSS**^**2**^	**MSI-H**^**2**^	P-value	**MSS**^**2**^	**MSI-H**^**2**^	Fold-change (p-value)
*EGFR*	0.62	0	0.0003				926+1088	369+197	2.51 (0.106)
*EXT1*	0.69	0.06	0.00099				829+285	618+244	1.34 (0.053)
*GNAS*	0.54	0	0.0011				16843+4876	13486+4555	1.25 (0.082)
*HOXA11*	0.46	0	0.00361				ND^3^	ND^3^	-
*HOXA13*	0.46	0	0.00361				368+364	496+318	0.74 (0.346)
*HOXA9*	0.46	0	0.00361				1979+2078	2521+1705	0.79 (0.472)
*IKZF1*	0.46	0	0.00361				ND^3^	ND^3^	-
*JAZF1*	0.46	0	0.00361				153+128	145+43	1.05 (0.847)
*LHFP*	0.54	0.06	0.0097				553+486	355+172	1.56 (0.202)
*MAFB*	0.62	0	0.0003				648+687	711+351	0.91 (0.776)
*TOP1*	0.54	0	0.0011				156+49	151+40	1.03 (0.768)
*MALT1*				0.69	0	0.00007	258+79	334+105	0.77 (0.051)

^1 ^The list of cancer gene from the Cancer Genome Project (http://www.sanger.ac.uk/genetics/CGP/).

^2 ^The sample sizes of MSS and MSI-H are 13 and 16, respectively.

^3 ^ND: Genes are not expressed in tumor tissue.

Among 24,526 annotated RefSeq transcripts (18,631 unique genes) of Illumina Human Ref-8 gene expression array, 12,012 (48.9%) were expressed in tumor tissues. 599 and 724 transcripts showed higher- or lower-expressions, respectively, in MSS tumors compared to MSI-H (Additional File 4). The transcript profiles of nine genes, as shown in Additional File 5, can be used to well classify CRC microsatellite status in clinical patients from Caucasian population [30]. Six of them showed concordant expression profiles between Caucasian and Han Chinese populations, but lower-expressed SFRS6 and higher-expressed SET genes of CRC MSS tumors in Caucasian were not found in Han Chinese, implying there are subtle population diversities in CRC transcript profiles.

Although there were numerous genes affected by CN gains and/or losses in CRC cancer genome, especially in MSS cases, some might not directly contribute to the levels of gene expressions. The patterns of differentially-expressed genes between CRC subtypes (two sample *t*-test with p-value < 0.05) are similar to those of CNA analysis at genome-wide scale (Figure 5b). Only 514 of 1,515 showing CNA frequency differences between subtypes were expressed in tumor tissue, and 271 of them (52%) were differentially expressed (p-value < 0.05, Additional File 6), suggesting the CN variations of genes might underline the expressional diversities between CRC MSS and MSI-H subtypes. For example, CN gains of *CPNE1 *genes were found in 8 of 13 MSS but not in MSI-H cases (Additional File 7), and the average *CPNE1 *expressional levels of MSS tumors was higher then that of MSI-H (1797.9 ± 879.5 *vs*. 963.3 ± 333.7, p-value = 0.008). *CPNE1 *gene showed the most significant correlation between CNAs and transcript levels (correlation coefficient, *r*^2 ^= 0.7). *CPNE1 *gene regulates tumour necrosis factor-alpha receptor signaling pathway and is over-expressed in liver cancer [31,32], but is still poorly investigated in CRC tumorigenesis.

## Discussion

This is a large-scale sporadic CRC study in an Asian population, and our results showed that the clinicopathologic features of MSI-H tumors were right-sided predominant, poorly or mucinous diffenentiated, less advanced disease and female predominant. Similar to previous studies with Lynch syndrome [6,22], MSI-H in our case series of sporadic CRC bear epigenetic change of *MLH1 *gene. However, the clinical features are distinctly different, and they tend to have older age onset of cancer and female predominant. For rectal cancer, the percentage of MSI-H and *MLH1 *methylation was only 2.8% (9/401) and 1% (4/401) respectively. On the other hand, right-sided colon cancer had, 16.3% and 11.2% MSI-H and *MLH1 *methylation, respectively. Therefore, dysfunction of MMR proteins might play different roles in the tumorigenesis of colon cancer *vs*. rectal cancer. It is noteworthy that all 22 samples with a *BRAF *(V599E) mutation were *MLH1 *hypermethylated, whereas 29 of 51 tumors with *MLH1 *hypermethylation did not have a *BRAF *mutation. These findings suggest that *MLH1 *hypermethylation might be an early event, occurred prior to *BRAF *mutation during CRC tumorigenesis.

We have applied high-density SNP array to detect copy number changes in the CRC cancer genome in the Taiwanese population, and compared the CNA frequencies between MSS and MSI-H subtypes. Previous CRC CN analyses primarily concerned with the Caucasian genetic backgrounds and these studies were hampered by the low-resolution of CGH array. Although different populations and technological resolutions were used in this study, the overall CNV pattern was globally similar to those from previous studies, indicating the mechanism of CRC tumorigenesis of different ethnic populations might be similar. Although EGFR CN gains were commonly found in MSS tumors (64%), some MSI-H tumors (14%) carried three or four gene copies. Previous studies have shown a small proportion of MSI-H tumors harbor multiple CNAs and chromosome abnormalities [17]. Consistently, we also observed some MSI-H tumors carried more than 1 Mb CNAs (Additional File 1), and 27.5% MSI-H tumors showed DNA aneuploidy. Studies showed that response predictors for CRC patients using cetuximab, EGFR monoclonal antibody, included *K-ras/Braf *mutation and *EGFR *gene CN, etc [33,34]. Further investigations are needed to clarify whether MSI tumors might be resistant to cetuximab for possible *BRAF *mutation or relatively low copy number of *EGFR *gene. Among 12,012 tumor-expressed transcripts, 514 genes showed significant CN gains or losses in MSS tumors, but 48% of them were not directly correlated with their expressional levels. For example, 8/13 MSS and 0/16 MSI-H tumors have *EGFR *CN gains; the expression fold-change of MSS/MSI-H group was 2.5 (962.4/368.8) but not significant (p-value = 0.10), caused by large standard deviation of *EGFR *expression levels (Table 2). Besides CNVs, other genomic variants, including SNPs and Indels, and epigenomic modifications all can regulate transcript levels, so an integrated analysis are needed to interpret the transcript diversities between CRC subtypes.

The identified CRC subtype-specific CN-altered genes should be seriously considered when investigating the mechanism of heterogeneous CRC tumorigenesis, and might be used as candidate markers in the drug therapy studies. The major discrepancy, and argument, between our results and other studies was that the proportion of MSI-H in our study was only 6.4%, lower than that of previous reports [35-38]. Selection bias and racial and/or environmental factors might affect the MSI incidence in CRCs. Because rectal cancer is less likely to show MSI-H than colon cancer [39], a lower rate of MSI-H colorectal cancer will be reflected in population-based studies. In studies without selection [39-41] incidence of MSI would be similar to our results.

## Competing interests

The authors declare that they have no competing interests.

## Authors' contributions

CHL, JKL, SCC, SFT and WSC conceived of experiments; CHL, SCC, YHC and HMC performed experiments; CHL, JKL, LHL and YTC provided and analyzed data; all authors read and approved the final manuscript.

## Supplementary Material

Additional file 1 **The size distribution of copy number variation in colorectal cancer**. CNVs were called by using Affymetrix Genotyping Console program based on the intensity data of Affymetrix SNP 6.0 array, and 20-probe criterion was used to filter out false-positive predictions. The sizes of identified CN changes from MSS CRCs were majorly between 50 and 500 kb, and a quarter of these alterations were smaller than 100 kb. Click here for file

Additional file 2 **Genes showing copy number (CN) differences between MSS and MSI-H CRC cases**. 1,515 genes were found to have CN frequency difference between MSS and MSI-H tumors using Fisher's exact tests (p-value < 0.05). Click here for file

Additional file 3 **The verification of EGFR copy number states of 48 CRC MSS and 48 MSI-H clinical samples**. qPCR approach was used to determine the *EGFR *CN states of 48 CRC MSS and 48 MSI-H samples. Click here for file

Additional file 4 **Differently-expressed transcripts between MSS and MSI-H CRC cases**. Among 24,526 transcripts of Illumina Human Ref-8 gene expression array, 599 and 724 transcripts showed higher- or lower-expressions, respectively, in MSS tumors compared to MSI-H. Click here for file

Additional file 5 **Expression fold-changes between CRC subtypes in different populations**. There were subtle diversities in CRC transcript profiles between Caucasian and Han Chinese populations. Click here for file

Additional file 6 **The combined analysis of copy number alterations (CNAs) and gene expressions**. 1,515 genes showing different CNA frequencies between CRC subtypes, and 514 of them were expressed in these tumor tissues. 271 of 514 genes (52%) show differential expressions between CRC MSS and MSI-H subtypes (two sample *t*-test with p-value < 0.05). Click here for file

Additional file 7 **The positive correlation between copy number and expression in *CPNE1 *gene**. The average *CPNE1 *expressional levels of MSS group was higher then that of MSI-H group (p-value = 0.008), and the gene CNs were highly correlated to expressional levels (liner regression correlation coefficient, *r*^2 ^= 0.7). Click here for file
