# Supplementary material for: Molecular profile and copy number analysis of sporadic colorectal cancer in Taiwan
Source: J Biomed Sci. 2011 Jun 7;18(1):36. doi: 10.1186/1423-0127-18-36 (PMC3123622; doi:10.1186/1423-0127-18-36)

**Additional File 1**. The size distribution of copy number variation in colorectal cancer. CNVs were called by using Affymetrix Genotyping Console program based on the intensity data of Affymetrix SNP 6.0 array, and 20-probe criterion was used to filter out false-positive predictions. The sizes of identified CN changes from MSS CRCs were majorly between 50 and 500kb, and a quarter of these alterations were smaller than 100kb.


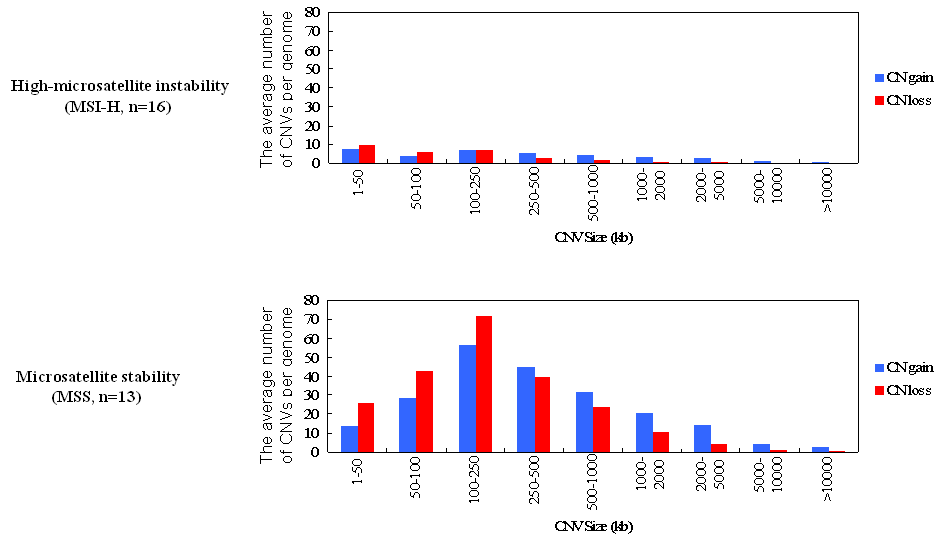

Supplement: Additional file 1 — The size distribution of copy number variation in colorectal cancer. CNVs were called by using Affymetrix Genotyping Console program based on the intensity data of Affymetrix SNP 6.0 array, and 20-probe criterion was used to filter out false-positive predictions. The sizes of identified CN changes from MSS CRCs were majorly between 50 and 500 kb, and a quarter of these alterations were smaller than 100 kb. [file 1423-0127-18-36-S1.DOC]
