# Supplementary material for: Molecular profile and copy number analysis of sporadic colorectal cancer in Taiwan
Source: J Biomed Sci. 2011 Jun 7;18(1):36. doi: 10.1186/1423-0127-18-36 (PMC3123622; doi:10.1186/1423-0127-18-36)

**Additional File 3**. The verification of EGFR copy number states of 48 CRC MSS and 48 MSI-H clinical samples. qPCR approach was used to determine the *EGFR* CN states of 48 CRC MSS and 48 MSI-H samples. Blue and green colors represent the clinical CRC MSS and MSI-H samples, respectively. The CN gain frequency of the independent CRC MSS group was 64.6% (31 of 48) and higher than 18.8% of CRC MSI-H subtype (n=48).


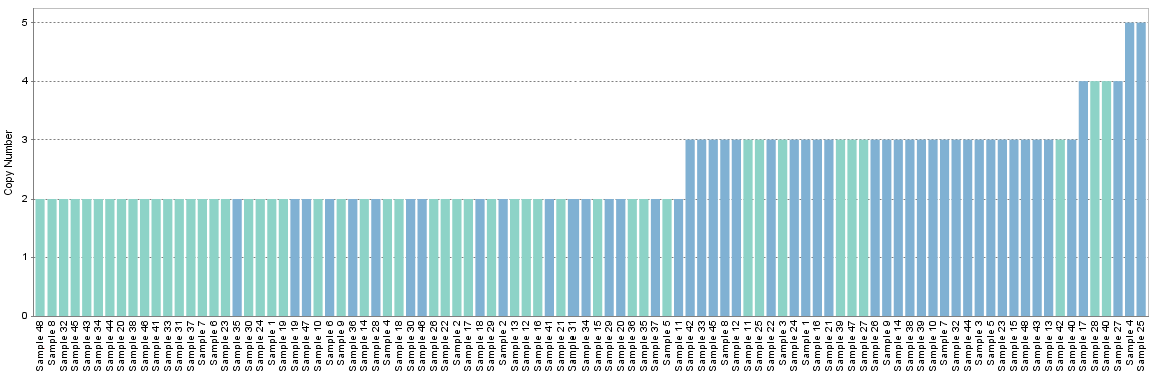

Supplement: Additional file 3 — The verification of EGFR copy number states of 48 CRC MSS and 48 MSI-H clinical samples. qPCR approach was used to determine the EGFR CN states of 48 CRC MSS and 48 MSI-H samples. [file 1423-0127-18-36-S3.DOC]
