# Supplementary material for: Molecular profile and copy number analysis of sporadic colorectal cancer in Taiwan
Source: J Biomed Sci. 2011 Jun 7;18(1):36. doi: 10.1186/1423-0127-18-36 (PMC3123622; doi:10.1186/1423-0127-18-36)

**Additional File 6**. The combined analysis of copy number alterations (CNAs) and gene expressions. 1,515 genes showing different CNA frequencies between CRC subtypes, and 514 of them (34%) were expressed in these tumor tissues. Among these 514 genes, 271 of 514 genes (52%) show differential expressions between CRC MSS and MSI-H subtypes (two sample *t*-test with p-value<0.05).


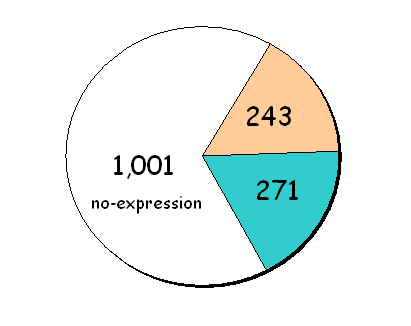

Supplement: Additional file 6 — The combined analysis of copy number alterations (CNAs) and gene expressions. 1,515 genes showing different CNA frequencies between CRC subtypes, and 514 of them were expressed in these tumor tissues. 271 of 514 genes (52%) show differential expressions between CRC MSS and MSI-H subtypes (two sample t-test with p-value < 0.05). [file 1423-0127-18-36-S6.DOC]
