# Supplementary material for: Molecular profile and copy number analysis of sporadic colorectal cancer in Taiwan
Source: J Biomed Sci. 2011 Jun 7;18(1):36. doi: 10.1186/1423-0127-18-36 (PMC3123622; doi:10.1186/1423-0127-18-36)

**Additional File 7**. The positive correlation between copy number and expression in *CPNE1* gene. CN gains of *CPNE1* genes were found in eight of 13 MSS cases (seven cases with 3 copies; one case with 4 copies), and none of 16 MSI-H cases had *CPNE1* gene CN gains. The average *CPNE1* expressional levels of MSS group was higher then that of MSI-H group (1797.9±879.5 *vs.* 963.3±333.7, two sample t-test with p-value=0.008), and the gene CNs were highly correlated to expressional levels (liner regression correlation coefficient, *r*^2^=0.7).


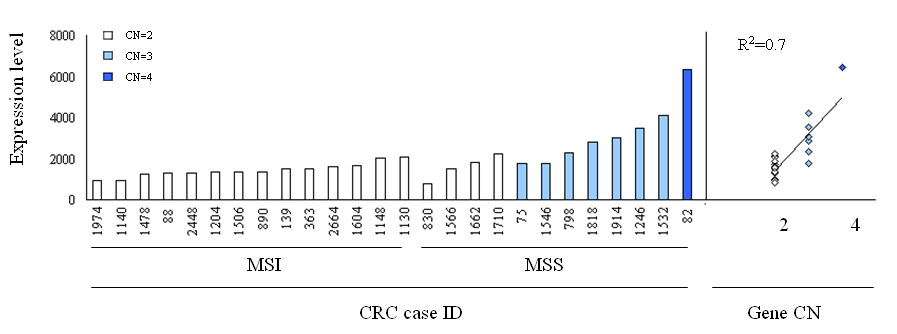

Supplement: Additional file 7 — The positive correlation between copy number and expression in CPNE1 gene. The average CPNE1 expressional levels of MSS group was higher then that of MSI-H group (p-value = 0.008), and the gene CNs were highly correlated to expressional levels (liner regression correlation coefficient, r2 = 0.7). [file 1423-0127-18-36-S7.DOC]
